# The Impact of the COVID-19 Pandemic on Social Workers at the Frontline: A Survey of Canadian Social Workers

**DOI:** 10.1093/bjsw/bcab158

**Published:** 2021-07-27

**Authors:** Rachelle Ashcroft, Deepy Sur, Andrea Greenblatt, Peter Donahue

**Affiliations:** Factor-Inwentash Faculty of Social Work, University of Toronto, Toronto, ON M5S 1V4, Canada; Ontario Association of Social Workers, Toronto, ON M5G 1Z8, Canada; Factor-Inwentash Faculty of Social Work, University of Toronto, Toronto, ON M5S 1V4, Canada; School of Social Work, King’s University College, Western University, London, ON N6A 2M3, Canada

**Keywords:** COVID-19, professional association, social work, survey, virtual care, well-being

## Abstract

Social workers are facing increasingly complex client needs during the coronavirus disease of 2019 (COVID-19) pandemic. Because of the social distancing requirements of the pandemic, social workers have undergone transformative changes in practice with the rapid uptake of virtual technologies. The objective of our study was to understand the experiences of social workers during the first-wave of the COVID-19 pandemic. We conducted a cross-sectional, web-based survey, comprised of close-ended and open-ended questions. Survey participants included social workers who were the members of a provincial social work association in Ontario, Canada. With *n* = 2,470 participants, the response rate was close to 40 per cent. Descriptive statistics were conducted on the close-ended questions. Two open-ended questions were coded using the thematic analysis. Nine themes were identified on the impact to social worker’s employment status: increased work-load; loss of employment; redeployment to new settings; early retirement; concern for personal health and safety; social workers in private practice seeing fewer clients; personal caregiving responsibilities; limiting recent graduates’ employment potential and social workers experiencing new opportunities. There were five themes on the impact on social work practice: clients with increasing complexities; challenges with transition to virtual care; benefits with transition to virtual care; adapting in-person services and personal well-being.

## Teaser

Social workers in Canada are experiencing insecure employment and difficulties in practice during the COVID-19 pandemic.

## Introduction

Social workers across all practice sectors are witnessing the devastating individual, interpersonal and societal impacts of COVID-19 (Banks *et al.*, 2020). People across the globe have experienced job loss, financial impacts and exacerbated mental health concerns (Abrams and Szefler, 2020). Social workers and other front line workers have been called to action to address the range of psycho-social crises emerging from the pandemic (Amadasun, 2020; Bern-Klug and Beaulieu, 2020; Peretz *et al.*, 2020). Social work practice brings an expertise grounded in a person-in-environment perspective; a systemic understanding of institutions and policies; an expertise in the social determinants of health and professional skills to support individuals, groups and communities (Skeketee *et al.*, 2017; NASW, 2020a). More than ever, there is an urgent need to ensure that social workers are well supported to respond to clients’ overwhelming psycho-social needs emerging during pandemic (Bern-Klug and Beaulieu, 2020; Walter-McCabe, 2020a,b).

### The role of social work during the COVID-19 pandemic

Social work harnesses a range of skills and competencies of importance to the emerging needs of the pandemic including risk assessments, crisis management, advanced care planning, individual and group therapy, case management, systems navigation, problem solving, resource allocation, community mobilisation and policy development (Skeketee *et al.*, 2017; Ashcroft *et al.*, 2018; Bern-Klug and Beaulieu, 2020; Walter-McCabe, 2020a). Recent scholarship illustrates how crucial the role of social work is to the current crisis (Walter-McCabe, 2020a,b). Social workers work across micro, mezzo and macro levels of practice and have been visible in fighting for social justice, particularly against xenophobia and racism, which has risen tremendously during the pandemic (Miller and Lee, 2020; Walter-McCabe, 2020a). Recognition of social work’s key competencies has resulted in calls for their increased involvement in policy-level decisions to attend to broader systems and social determinants of health related to the pandemic (Banks *et al.*, 2020; Truell, 2020; Walter-McCabe, 2020a). Truell (2020) highlighted the key social work practices and advocacy initiatives for change enacted during the pandemic, such as focusing efforts to ensure that social services are accessible during periods of lockdown. Mental health difficulties have increased during the pandemic, as social isolation has both created and exacerbated mental health concerns (Usher, 2020). Adhering to standards of practice that involves specialised interventions to individuals, groups and families and by maintaining accessible service delivery, social workers are critical providers during the pandemic (Walter-McCabe, 2020a). Understanding how delivery of services has transformed is essential to support social workers during a period of rapid change.

### Rapid transformation to virtual care

The onset of the COVID-19 pandemic required social workers to quickly transform practice to adhere to public health guidelines—such as the use of personal protective equipment (PPE), adhering to physical distancing requirements and closure of non-essential in-person workplaces—whilst still maintaining connections with their clients (Alex *et al.*, 2020; Krelle *et al.*, 2020; Donnelly *et al.*, 2021). Prior to the pandemic, the use of synchronous virtual care—which refers to both telephone and video appointments—was minimal for many health and social service settings (Berzin *et al.*, 2015; Glauser, 2020). However, some social workers have used virtual care in clinical practice even prior to the pandemic for counselling, group therapy, case management and other activities (Marziali *et al.*, 2006; Reamer, 2013; Ramsey and Montgomery, 2014; Mishna *et al.*, 2019). Some social workers in rural and remote communities have long relied on telephone and video technologies, yet the uptake has not been widespread (Ramsey and Montgomery, 2014; Bryant *et al.*, 2018; Mishna *et al.*, 2019; Tadic *et al.*, 2020). Despite having some experience of using virtual care in practice there is a widespread gap in the education and training of social workers on how to effectively and ethically integrate virtual technology into practice (Mishna *et al.*, 2015; Berzin *et al.*, 2015). Until recent, the integration of virtual technologies in practice has remained limited (Berzin *et al.*, 2015). The pandemic has accelerated the use of virtual care in Canada, USA and elsewhere in the world (Glauser, 2020; Krelle *et al.*, 2020). Social workers have had to quickly integrate virtual care in practice to maintain continuity of client care despite having little training and access to technologies specifically developed for social work practice.

### The well-being of social workers during the COVID-19 pandemic

Knowledge concerning the impact on front line workers of trying to address increasingly complex clients’ needs through virtual care in the context of a pandemic is only just evolving (Williamson *et al.*, 2020). Emerging evidence indicates that health care workers are at risk of developing or exacerbating mental health issues during the pandemic (Greenberg, 2020). Front line workers are unable to deliver care as usual, yet are required to perform their already very challenging work in a much more constrained context (Greenberg, 2020; Williamson *et al.*, 2020; Donnelly *et al.*, 2021). Challenges faced by front line workers include a lack of resources to do their jobs, fear about their own health, guilt, shame, grief, exhaustion and a lack of guidance and training to navigate their changing roles, which put front line workers at risk of moral injury (Banks *et al.*, 2020; Williamson *et al.*, 2020; Donnelly *et al.*, 2021). Moral injury refers to the profound psychological distress resulting from actions and inactions that violate one’s ethical or moral code, and may result if staff feel unprepared for the consequences of decisions made and feel a lack of both social and leadership support (Williamson *et al.*, 2020). For non-medical front line workers, like social workers, not being designated as an essential worker in some places may also lead to insecure employment during a period of shifting human resourcing (Banks *et al.*, 2020). Banks *et al.* (2020) report that social workers have faced struggles prioritising clients’ increasingly complex needs with limited resources, navigating policies whilst balancing client needs. Social workers reported that they are struggling to manage daily work routines, meet client needs and combat the range of social justice issues inherent in the pandemic (Banks *et al.*, 2020). The COVID-19 pandemic context is one of the high client needs, high demands and transformative changes in practice, which may place social workers and other front line workers at risk of burnout (Bohman *et al.*, 2017).

### Rational for study

As part of a larger survey seeking to understand the needs of social workers in Ontario, Canada to determine how the Ontario Association of Social Workers (OASW) could best support social workers, we asked members of the OASW about the impact that the pandemic has had on them. The OASW is a provincial organisation that supports and advocates with and on behalf of social workers in Ontario. The objective of the larger survey was to explore how the Association could best support members. This goal became particularly important when the pandemic rapidly shifted practice and increased isolation amongst members. Our study sheds light on the experiences of social workers on the front lines during the pandemic and offers insights to better support them with the ongoing demands of the COVID-19 pandemic. Although the survey contained a range of questions related to membership supports, this article specifically presents the sub-set findings that help to understand experiences of social workers during the first-wave of the COVID-19 pandemic. To our knowledge, this is the first study to survey Canadian social workers for this purpose.

## Methods

### Study design

This was an exploratory study using a cross-sectional, online survey disseminated through Survey Monkey software to answer the question: How can the OASW better support social workers across Ontario? We opted for an online survey because it is an effective tool for gathering large amounts of data in a relatively short period of time (Albudaiwi, 2018). The overarching aim of the survey was to determine how OASW services and resources could best provide support to social workers across Ontario (i.e. topics for future educational training). This article presents qualitative findings from the subset of questions that answer the following question: What is the impact of COVID-19 on social workers in the province of Ontario, Canada? Our study conforms to internationally acceptable research and professional ethical guidelines. Research ethics approval was obtained from the University of Toronto (REB Protocol #38945).

### Study context

Ontario is Canada’s most populous province with 14.7 million residents (Government of Ontario, 2021a). On 17 March 2020 the government declared a provincial state of emergency, which required the closure of most indoor facilities and businesses except those providing essential services (Government of Ontario, 2020). Public Health Ontario (2020) reported a total of 34,911 cases of COVID-19 in Ontario near the end of the first wave of the COVID-19 pandemic (as of 28 June 2020), and 467,000 during the third wave (Government of Ontario, 2021b). At the time of our study, ‘Canada’s case-fatality rate (8.29%) was higher than that of both the global average (5.17%) and the United States (5.17%)’ (Yu *et al.*, 2020, p. 2).

Social workers are a regulated profession in Ontario, with approximately 20,000 social workers currently registered in Ontario (Canadian Institute for Health Information, 2021). In Ontario, social workers are employed in various settings including community health organisations, hospitals, schools, addiction and mental health treatment, child welfare, private practice and other locations (OASW, 2021). Many social-work positions are directly and/or indirectly funded through government. Some positions are funded by donations whilst some social workers work as independent practitioners in private practice (OASW, 2021).

The Ontario College of Social Workers and Social Service Workers (OCSWSSW) is the regulatory body for social workers in Ontario (OCSWSSW, 2019). On 20 March 2020, the OCSWSSW issued a memo, strongly recommending that members consider suspending all non-essential social work services until further notice (OCSWSSW, 2020). The OCSWSSW further suggested that members may wish to consider options for providing services by electronic means—through the use of any electronic device (including a computer, tablet, smartphone or landline) or any electronic format (including the Internet, social media, online chat, text, video or email) (OCSWSSW, 2020).

### Sample

We used a convenience sampling technique to engage a population of social workers. Potential participants were those who were able to complete an online survey in English, and a member of OASW. At the time of survey, 6,245 social workers were members of OASW. All OASW members have access to email and OASW uses an email Listserv to communicate regularly with members. The response rate was close to 40 per cent for a total of 2,470 participants.

### Data collection

We conducted the online survey from 9 June 2020 to 21 September 2020. All OASW members were sent an invitation to participate in the online survey through recruitment emails and posts on social media (e.g. Twitter). All responses were anonymous. There are advantages for collecting data using online surveys. For example, it is easy for an online survey to be distributed and circulated, thus increasing likelihood of reaching a wide sample (Lawrence *et al.*, 2016).

The survey consisted of fifty-one open-ended and closed-ended questions in total and took approximately 15 min to complete. The domains in the survey included respondent demographics, professional practice background, professional development needs, OASW membership service preferences, future direction of OASW and impact of COVID-19. Other than demographics, this article presents findings that draw from the section of the survey pertaining to the impact of COVID-19, which included two close-ended and two open-ended questions. The two close-ended questions were (i) ‘What was your primary mode of service delivery prior to COVID-19?’ and (ii) ‘Since the start of COVID-19, what is your primary mode of service delivery?’ The two open-ended questions were (i) ‘Please share how your social work practice has changed as a result of COVID-19’ and (ii) ‘Please describe any impact that COVID-19 has had on your employment status.’

The survey design was inspired by a previous provincial-wide survey of social workers (Ashcroft *et al.*, 2018). Two authors developed the initial survey tool and one author helped refine the final iteration. All research team members reviewed and revised questions for relevancy. Prior to implementation, we pilot tested the survey with three individuals who fell within our target population. Pilot testing, a survey, is recommended to ensure that questions are correctly worded, there is clarity in directions, and to ensure that skip patters work as intended (Thomas, 2004). We also conducted a cognitive interview to trial and improve the survey tool (Drennan, 2003). Cognitive interviewing required that an informed respondent with an expertise in social work completed the survey in the presence of the third author and was asked to think aloud whilst going through the survey (Drennan, 2003). We revised wording and order of some questions based on information we learned through the cognitive interviewing process.

### Data analysis

Descriptive statistical analyses were performed on the close-ended questions using Survey Monkey software. Thematic analysis was conducted on the sub-set of open-ended questions (Braun and Clarke, 2006). The qualitative data from the open-ended questions were downloaded into Excel and two authors analysed the data. Coding was conducted manually. Excel is an effective tool for analysing qualitative data collected in open-ended survey questions (Ose, 2016) and has successfully been used by the lead author on other survey projects (Ashcroft *et al.*, 2018; Donnelly *et al.*, 2021). Analysis occurred in the six steps outlined by Braun and Clarke (2006). First, the coders became familiar with the data by reviewing the data. Second, the two authors generated initial codes in the data. One author acted as the primary coder whilst another acted as the secondary coder. When the initial coding was complete, the two coders met to review the coding structure. Third, the codes were reviewed for potential themes. Fourth, the research team reviewed the themes. Fifth, the themes were named, defined and refined. Finally, the themes and codes were related back to the study aims through the process of manuscript preparation. Exemplar quotes were identified during the analysis process.

## Results

### Demographics

Although participants spanned a range of ethnicities, most participants identified as Caucasian (89 per cent) and female (83 per cent). Participants spanned all five provincial geographical regions including Central East, Central West, Eastern, Northern and Western (Ministry of Health 2019). Most participants (83 per cent) held a Master of Social Work (MSW) degree, some (9 per cent) held a Bachelor of Social Work, some (3 per cent) had a PhD in social work whilst others (5 per cent) were students in process of completing a social work degree. See Supplementary Table S1 for an overview of participant demographics.

### Social work practice experience

There was a broad range of social work practice experience represented amongst the participants: some (33 per cent) were in practice for ten years or less, some (24 per cent) were in practice between eleven and twenty years, whilst other (41 per cent) participants had been in social work practice for more than twenty-one years. In terms of employment status, most participants (53 per cent) were in full-time permanent positions whilst other participants held part-time, temporary or casual positions. See Supplementary Figure S1 for details on participant employment status. Further, participants practiced in a broad range of institutional settings with the majority (37 per cent) working in private practice, followed by health care (19 per cent). See Supplementary Table S2 for the types of institutional settings within which participants were practicing. Many of those who selected ‘other’ indicated that they worked in primary care.

Participants worked with clients across the lifespan, including children under thirteen (5 per cent), youth ages thirteen–eighteen years old (9 per cent), young adults ages nineteen–twenty-nine (10 per cent), adults ages thirty–sixty-four (41 per cent), and seniors aged sixty-five and up (9 per cent) who are one of the most vulnerable population in COVID-19. A number of participants (20 per cent) indicated that their client population spanned all age groups. This question was not applicable to a small number of respondents (6 per cent), which were likely those social workers who held indirect practice positions, such as leadership or policy. When asked their area of specialisation, participants identified a broad range of specialty areas with mental health being the most frequent (51 per cent) response.

### Mode of service delivery

There were *n* = 2,148 participants who responded to the question ‘What was your primary mode of service delivery prior to COVID-19’. As well, there were *n* = 2,133 participants who responded to the question ‘Since the start of COVID-19, what is your primary mode of service delivery?’ There was a dramatic change in the types of modalities used by social workers to provide direct client care after the start of the COVID-19 pandemic. Prior to the pandemic, participants reported that most (87 per cent) client services were in-person. Following the provincial declaration of emergency on 17 March 2020 (Government of Ontario, 2020) that required closure of non-essential in-person workplaces, virtual (telephone and video) appointments became the most frequent (76 per cent) modality for providing client services. A small number (10 per cent) of participants continued to provide in-person care.

### Impact of COVID-19 on social workers’ employment status

There were *n* = 2,091 participants who provided a response to the open-ended question: ‘Please describe any impact that COVID-19 has had on your employment status’. The nine themes identified include (i) increased work-load; (ii) loss of employment; (iii) redeployment to new settings; (iv) early retirement; (v) concern for personal health and safety; (vi) social workers in private practice seeing fewer clients; (vii) personal caregiving responsibilities; (viii) limiting recent graduates’ employment potential and (ix) social workers experiencing new opportunities.

#### Increased work-load

Many participants reported having experienced an increased work-load resulting from the COVID-19 pandemic. One of the reasons for increased work-load was due to working longer hours. One participant reported, ‘To be honest, I feel as though I have had to work more as a result of COVID-19’. Another participant stated, ‘It resulted in higher workload and extensive overtime hours’ whilst a third participant reported, ‘[in] the first few months my workload ramped up’. Additionally, another participant similarly reported, ‘My caseload has more than doubled’.

#### Loss of employment

Many participants reported that their employment came to a halt for a number of reasons related to the COVID-19 pandemic. One participant stated that, ‘I was left unemployed by COVID-19’ and another reported that employment, ‘stopped in its tracks’, whilst a third participant stated, ‘I have had to stop working in [Long Term Care] temporarily due to provincial directive’. One participant explained that they were laid off from their employment, ‘[I was] laid off due to issues with my internet connection [and am] currently on [Employment Insurance] for the first time ever’.

#### Redeployment to new settings

Some participants reported that they were re-deployed from their usual place of employment to alternate settings. For example, one participant stated, ‘Fortunately the hospital filled my hours by redeploying me temporarily to assist [occupational] health’. Another participant reported, ‘My job changed completely to a new job. Everything has changed’. Redeployment to new settings meant that some participants have experienced substantial change in their professional roles. According to one participant, ‘It’s pretty dismal now. I have been relocated, have different days and hours. I’ve lost my office. I’ve lost a direct telephone line … I can’t utilize therapeutic interventions that I used to. I feel professionally neutered’.

#### Early retirement

Some participants explained that they entered early retirement because of COVID-19. For example, one participant stated ‘[COVID-19] pushed me into retirement because my treatment style does not translate [online]’. Similarly, another participant stated that, ‘It hastened my retirement. I am still working but minimally’. A third participant also followed a similar path, ‘I have fully retired as a result of the pandemic, with the exception of volunteer phone counselling’. Another participant emphasised that their choice for retirement was related to the shift to virtual care: ‘I retired. [I’m] not able/willing to work in a “virtual” context. I did try, but it never felt real or alive. Clients would have been willing, but the mechanicals, for me, stripped the heart out of the process’.

#### Concern for personal health and safety

Some participants experienced job loss and/or disruption to their employment due to concern for their own personal health and safety. For example, one participant explained, ‘[I] left job at hospital due to risks to my age demographic [and am] unsure if I will return’. A second participant reported, ‘[I am] on leave due to stress’. Similarly, a third participant stated, ‘I’m on a one-month stress leave, largely brought on by the stress from the initial phase of the pandemic’. One participant explained that employment came to a halt because they contracted COVID-19, ‘I actually got COVID-19 so recovery and illness took me away from work’.

#### Social workers in private practice seeing fewer clients

Participants in private practice reported that they saw fewer clients since the onset of the COVID-19 pandemic. For example, one participant stated that COVID-19 ‘decimated my practice’ whilst another participant stated, ‘I have a few of my pre-COVID-19 clients but no new clients. My private practice is almost dead’. A third participant agreed by saying, ‘private practice slowed down, loss of income’. One participant explained, ‘the number of clients has decreased [because] some of my clients prefer in-person treatment’.

#### Personal caregiving responsibilities

Some participants reported that their employment came to a halt because of the demands related to caregiving responsibilities. As described by one participant, ‘[I] had to stop work to stay home caring for 3 young children under the age of 5’. Another participant explained, ‘[I took] a couple months off work due to the virus as a caregiver’.

#### Limiting recent graduates’ employment potential

Participants who were students and/or recent graduates raised concerns that the disruption to their internships created barriers with securing new employment. For example, one participant explained: ‘I am a MSW student who was unable to achieve a clinical placement because of COVID and had to take a remote research placement. I am now leaving the MSW programme with little to no clinical skills and am extremely concerned how this will affect job prospects because I am lacking very important skills’. Recent social work graduates expressed concerns that the limited internship training would diminish their future employment prospects. One participant stated, ‘Potential employers have expressed concern about new social workers experience and skill level in working directly with clients due to limited practicum hours’. Another recent graduate agreed and stated, ‘Prospective employers have expressed concern about students’ readiness for field work with limited direct client experience’. Lastly, another participant noted: ‘[COVID-19] has likely prolonged my unemployment status, as I was in the interview process for a couple of jobs and they were cancelled’.

#### Social workers experiencing new opportunities

Some participants noted that they had new employment opportunities that emerge during the pandemic. For example, one participant reported, ‘I’ve got an 8-week contract that I wouldn’t have otherwise had as a result of COVID-19’. In addition, some participants explained that new opportunities emerged in the expansion of existing social work roles. One participant explained that the pandemic ‘expanded some roles of social workers’. According to another participant, ‘I have witnessed a shift these last few months in which the act and practice of social work was so relied upon’.

### Impact of COVID-19 on social work practice

There were *n *= 1,370 participants who provided a response to the open-ended question: ‘Please share how your social work practice has changed as a result of COVID-19’. The five themes identified include (i) clients with increasing complexities; (ii) challenges with transition to virtual care; (iii) benefits with transition to virtual care; (iv) adapting in-person services and (v) impacting personal well-being.

#### Clients with increasing complexities

The two types of complexities were: (i) mental health and addictions and (ii) intimate partner violence. First, participants noted that they were seeing clients with a range of mental health challenges. According to one participant, ‘my clients’ needs are much higher and they have been struggling a great deal’. Similarly, a second participant explained, ‘My practice has become busier [with] more crisis situations [such as] suicidal ideation, severe depression’. Another participant also explained having an increased demand for mental health services, ‘Increased demand … and distress increased for mental health supports’. Another participant noted, ‘I have noticed that clients’ mental health and addictions have been exacerbated during this pandemic’ resulting with a higher ‘complexity of casework’.

Second, participants noted that they were seeing more clients in situations of intimate partner violence. One participant reported, ‘Intimate partner violence has been on the rise. Practicing from a trauma informed lens has been crucial for myself as a social worker and clients’. Another social worker shared that: ‘Domestic violence has been massively overlooked … I’ve felt a lot more uncertain about how to navigate these issues’.

#### Challenges with transition to virtual care

*Learning curve*. When asked to describe the impact that the COVID-19 pandemic has had on them, participants overwhelmingly reported a range of challenges they encountered with the implementation of remote virtual care. A key challenge reported by participants was the initial learning curve of transitioning to virtual care ‘New learning—we are open and serving individuals virtually or by telephone’. Another participant agreed and stated that, ‘[It is a] huge learning curve becoming familiar and competent with virtual services’. Further, another participant expressed concerns because of the, ‘challenges transferring to online with little direction’ in the early days of the pandemic. However, according to one participant it, ‘just demonstrates that as social workers being flexible, creative and adaptive are core practice skills’.*Lack of technological infrastructure*. The ability to transition to virtual care was difficult across some geographical regions. One challenge faced by participants in some rural and remote communities was the lack of technological infrastructure to support consistent high-speed Internet. One participant explained, ‘The lack of consistent internet across the province also has a negative impact’. Another participant agreed, ‘Videoconferencing is better than the phone but is not always available and reliable in the north’.

*Decreased access for some client populations.* Participants indicated that virtual care created access barriers for (i) children; (ii) homeless populations and (iii) some older adults. First, participants described various challenges when providing services for children. As one social worker mentioned, ‘As a child therapist, it has made it very difficulty to remotely engage little children under eight years of age in a remote session hence the quality of work has suffered’. For this reason, another social worker who worked directly with children prior to the pandemic, shifted to working with the parents instead. Second, participants noted that homeless populations were unable to access virtual services. One participant stated, ‘I have not had regular contact with the homeless’. Another participant expressed concerns with the lack resources available for homeless populations during the pandemic, particularly since those that are homeless lack ‘access to the technology required for virtual service’. Third, some participants noted that virtual care created barriers for some older clients who were not ‘tech savvy’.

*Lack of face-to-face contact.* Participants indicated that the lack of face-to-face contact created difficulties with (i) conducting assessments and (ii) therapeutic alliance. First, many participants stated that conducting assessments was particularly challenging when providing services by telephone and/or video. One participant stated, ‘Face-to-face visits have been replaced by telephone or remote contact, making assessments extremely complex. This makes our work tougher and opens the door to errors and misses’. Second, the lack of face-to-face contact may create difficulties with the therapeutic alliance. One participant noted, ‘Virtual instead of face to face … diminishes somewhat the client–therapist alliance and ability to pick up on non-verbal cues’. Another participant stated, ‘[It is] hard to do certain aspect of the job with little face-to-face’.

### Barriers implementing certain practice activities

First, participants indicated that some types of therapeutic modalities are inappropriate for virtual care. For example, ‘The other area of my practice is intrafamilial sexual abuse—the subtleties of the work simply cannot, nor should not, be done via a virtual platform. The impact of COVID-19 is frightening for individuals who relied on services they can no longer receive’.

Second, providing group interventions online creates confidentiality challenges. For example, one participant noted, ‘We will be doing things online, but I am concerned about how mutual aide will be achieved in the groups. Confidentiality is also a concern’.

Third, doing community engagement work was considered more difficult to do virtually.

A participant explained, ‘The impact on engagement and relationship building is significant and both require more time and different strategies. Services that include stakeholder consultations and facilitating community are online also require more and different upfront planning and delivery’.

#### Benefits with transition to virtual care

*Facilitating access.* Some participants indicated that transitioning to virtual care made services more accessible for some clients. One participant explained, ‘This has been beneficial for some clients [because it is] easier to access to care’. Another participant held a similar opinion, ‘Moving to virtual practice … expanded the ability to meet the needs of clients’.

*Enhancing some treatment modalities*. Some participants spoke of the benefits of virtual care for some types of treatments. For example, one participant reported, ‘It has opened a great deal of opportunity that I would not have considered before, including conducting Exposure and Response Prevention for OCD in … people’s houses. In this way, I think some treatments have been enhanced as a result of online virtual services’.

#### Adapting in-person services

The two sub-themes of adapting in-person services are (i) using PPE and (ii) meeting clients outdoors. First, some participants working in settings that continued to operate onsite and in-person described needing to incorporate new safety standards to protect themselves and clients from contracting the coronavirus. One participant explained that ‘PPE is required when meeting all clients’. Yet PPE created challenges for some social workers. A participant elaborated, ‘PPE makes interpersonal connection and reading of body language limiting on many levels … this is the new norm’.

Second, because of restrictions on non-essential in-person workplaces, some social workers were creative and met clients in outdoor settings. One social worker noted, ‘I also find that I have become much more creative in working to integrate … social distance yet still have in-person sessions … outdoor meetings at a distance in private locations … where there is privacy to talk without fear of breaches in confidentiality but where in-person dialogue can happen’.

#### Impact on personal well-being

*Positive impacts*. Some participants described positive impacts on their well-being including having ‘flexible [work] hours’, time saved on not having to commute to the office, and finding that they were able to have a better work–life balance. A second participant reported, ‘I get to work from home and pay down some debt because my expenses in some ways have decreased’. Another participant stated, ‘All services are virtual care and are provided from home setting, as per directive from employer … .[I] appreciate [the] flexibility inherent in this’. Additionally, some participants described how they were able to empathise with clients because they were sharing a similar contextual experience, ‘Adapting to technologically mediated work and the boundary challenges that sharing many of the same stressors that clients bring to session has created has been difficult, but interesting. It’s made me a better, though more exhausted, therapist’. *Negative impacts.* Participants overwhelmingly expressed concerns about the emotional impact on their well-being. First, many participants reported that they had an increase in work demands with little additional compensation. ‘COVID-19 has doubled/tripled my workload resulting in working late into the night without expectation of compensation. This is true for myself, my colleagues, and most social workers I have connected with. We are expected to work at a 110 per cent during a global pandemic with little to no resources or support’. Additionally, another participant explained, ‘there has been no additional compensation for being a frontline worker meeting clients in-person throughout the duration of the pandemic’.

Second, the transition of working virtually from home had challenges for many. One participant explained, ‘Shifting to working from home was extremely difficult in terms of practice’. Another participant stated, ‘I have remained employed but the boundaries between home/work life have blurred’. Blurred boundaries between home and work lives have heightened stress for participants. ‘Having to be home to take care of my children and the various new stressors that COVID has presented has made me less competent, timely and responsive. It has caused a fair amount of distress and disengagement on my part that feels a little like “burn out”.’

Third, the emotional impact of the COVID-19 pandemic on social workers is profound. One participant explained the emotional impact of working in an acute-care setting, ‘Having to manage units with COVID-19 patients has been hard … I feel a bit traumatized and disillusioned. I’m not sure if I can return to my previous position or keep working in health care from a moral perspective. [I’m] kind of feeling tired/burnt out’. Another participant had a similar experience, ‘I am an ICU Social Worker and COVID has … the inherent health risk factor and generalized fear in serving this patient population [and] the physical and emotional toll on me as a frontline professional dealing with this unprecedented situation, the traumatic experiences, the associated higher death numbers’.

Many participants described fatigue related to the shift to virtual care. One participant stated, ‘the personal changes that come with so many online hours [is] in many ways, more tiring!’ Working virtually from home has left many participants feeling ‘very isolated’. Many participants described feelings of profound stress. One participant described feeling ‘demoralized’ whilst another was ‘on leave due to stress’. Additionally, participants reported mental health difficulties. For example, one participant explained, ‘Increased mental health stressors, no work life balance, increased anxiety from helping others’. Some participants reported feeling burnout. One participant stated that they felt ‘profound burnout’ whilst another elaborated, ‘I have never felt burn out in all my years of working but dealing with my Internet issues and subsequent unemployment caused burnout’.

## Discussion

Social workers are integral to assist with the range of psycho-social and mental health needs emerging during the pandemic (Amadasun, 2020; Holmes *et al.*, 2020; Walter-McCabe, 2020a,b). The COVID-19 pandemic has heightened client complexities (Abrams and Szefler, 2020; Walter-McCabe, 2020a). Our study demonstrates that social workers are seeing clients with increasing complexities related to mental health and addictions, and intimate partner violence. Social workers are essential during and beyond this crisis to respond to these emerging complexities (Walter-McCabe, 2020a). Ensuring sustainability of social workers to respond to the vast psycho-social needs associated with COVID-19 means we must be cognisant of the professional and personal impact that they are enduring in this pandemic. The current circumstances have required social workers to pivot but not without cost and alarm.

The pandemic has stimulated social innovation for social workers (Schiavo, 2015) by facilitating a rapid and broad uptake of synchronous virtual care—telephone and video appointments. In some ways, the pandemic has brought social work closer to the goals of integrating innovative technologies in practice (Berzin *et al.*, 2015). Online services can enhance access for persons with disabilities, those living with social anxiety and those residing in rural or underserviced areas (Mishna *et al.*, 2015; Jong *et al.*, 2019). Further, virtual care has made it possible for many social workers to continue providing services to clients even during a period of physical distancing (Walter-McCabe, 2020b). These benefits were noted in our study.

Our study, however, identified numerous challenges faced by social workers following the rapid transition to virtual care. One major barrier experienced by some of our participants had to do with limited access to high to stable high-speed Internet for those residing in rural and remote communities (Lints-Martindale *et al.*, 2018; Baylak *et al.*, 2020). Despite some improvements over the last decade (Jong *et al.*, 2019), achieving social work’s grand challenge to improve practice innovation (Berzin *et al.*, 2015) through technology requires further commitment by policy makers to ensure equitable access to consistent high-speed Internet access rural and remote geographical regions (Lints-Martindale *et al.*, 2018; Baylak *et al.*, 2020). Additionally, our participants have expressed great concerns about the adaptations of social work practice to virtual care so as not to negate the relational nature of social work practice. Other challenges noted in our study included barriers faced by some client populations, difficulties adapting some practice activities, and issues of confidentiality. Our study demonstrates that using virtual care in practice is new for many social workers. As virtual care continues, more guidance in the form of training and policies needed to bolster social work’s capacity for integrating technology in practice (Berzin *et al.*, 2015; Mishna *et al.*, 2017, 2019).

Despite the rapid uptake of virtual care, many social workers continue to provide in-person care during the COVID-19 pandemic. One of the worldwide challenges faced by health care workers during the first-wave of the pandemic was the availability of PPE (Rawaf *et al.*, 2020). Social workers provide front line services that warrant safety, precaution and personal protection. Our study shows that some social workers are using creative approaches and meeting with clients outside. However, little remains known about the experiences of social workers providing in-person care to those who have contracted the coronavirus.

Our study illustrates that social workers are enduring immensely challenging situations during the pandemic that are leaving personal imprints. There is a growing body of literature examining the shared realities of social workers and the clients they serve (Dekel and Baum, 2010; Pentaraki, 2017). A concerning finding from our study is that social workers’ employment is insecure. Similar to Pentaraki’s (2017) findings in Greece, some social workers in Canada may be experiencing a shared austerity reality with their clients during the COVID-19 pandemic. The economic impacts of the COVID-19 pandemic are unprecedented (Statistics Canada, 2020), and have been felt by social workers in our study.

Dekel and Baum (2010) provide a conceptual understanding that helps explain the personal impact on social workers who work in a shared traumatic reality with their clients. A shared traumatic reality exists when social workers and their clients are confronted by the same threat (Dekel and Baum, 2010), as is the case with the COVID-19 pandemic. Studies have demonstrated negative consequences for social workers practicing in a shared traumatic reality including experiencing emotional distress during and following the event, and feeling less competent and effective in their professional role (Dekel and Baum, 2010). Distress arises in part because the shared experience can blur professional boundaries and ignite feelings of helplessness as social workers deal with one’s own personal losses associated with the traumatic event (Dekel and Baum, 2010). A key positive impact that can arise for social workers embedded in a shared traumatic reality with their clients is the potential to have a greater understanding of clients’ emotions and thus respond in a more empathetic and effective way (Dekel and Baum, 2010), as was identified in our study.

It is imperative that we strengthen social workers’ circle of support in order to enhance the collaborative professional communities (Dekel and Baum, 2010). Front line workers require informal and formal supports to assist with the overwhelming emotional and mental health burdens they are experiencing (Williamson *et al.*, 2020). Organisational and professional leadership is essential at this time to acknowledge these experiences, embed necessary supports and advocate and inform institutional structures that support front line workers during and after the pandemic (Greenberg, 2020). Nurturing provider wellness rests on three major domains: personal resilience, efficiency of practice and a collaborative culture of support (Bohman *et al.*, 2017). Social workers are experiencing and adapting to a new normal and this includes increasing influence on well-being and community. In addition, the profession contributes to the fabric of societal functioning and caring across generations. By calling attention to the support needs of social workers, our study aims to build social workers’ capacity so that we can continue to help repair and support those clients most affected by the COVID-19 pandemic.

### Limitations

First, findings from our study may not be applicable to all social workers. We conducted this study on social workers in Ontario, Canada. Social workers’ experiences during the pandemic may vary across provinces, countries and other geographical locations. This is in part because infection rates and lockdown rules varied across geographical locations. In addition, social workers are not homogenous in their experiences because there are a range of different type employers, organisational mandates and access to benefits such as time off with sick pay.

Second, our study did not explore the unique experiences of racialized social workers who have continued to care for clients and communities during a period of heightened xenophobia and racism (Miller and Lee, 2020; Walter-McCabe, 2020a). Our study relied on a convenience sample; therefore, an analysis of this was not possible from our data. Our survey did not directly ask social workers about their experiences with racism during the pandemic.

Third, our study occurred during the first-wave of the pandemic and we anticipate that experiences of social workers will continue to evolve as the pandemic progresses. Lastly, a limitation of online surveys relates to the potential that some participants may not complete all questions (Albudaiwi, 2018).

## Conclusion

The current pandemic has had far-reaching impacts, including social workers and the people and communities they serve. Not only have we seen an increased demand for our services, the complexity of the issues facing our clients has grown exponentially. Despite the many challenges presented by the pandemic as to how we deliver services, social workers have continued to provide essential services to individuals, families and communities. In addition, social workers continue to focus on improving conditions of daily living in a changing environment. Social workers have rapidly adapted to virtual care and have integrated innovative technologies in practice in an astounding way. Whilst social workers have seen some positive outcomes in innovative approaches for service delivery, social workers have also experienced personal and professional burdens. Stress, fatigue and burnout are just some of the many costs of caring incurred by social workers. We need to nurture collaborative professional communities, now more than ever, to ensure the well-being of social workers for the duration of the pandemic and beyond.

## Supplementary material

Supplementary material is available at *British Journal of Social Work Journal* online.

## Supplementary Material

bcab158_Supplementary_Data
