# Supplementary material for: The Impact of the COVID-19 Pandemic on Social Workers at the Frontline: A Survey of Canadian Social Workers
Source: Br J Soc Work. 2021 Jul 27:bcab158. doi: 10.1093/bjsw/bcab158 (PMC8406887; doi:10.1093/bjsw/bcab158)
Supplement: bcab158_Supplementary_Data [file bcab158_Supplementary_Data.zip › Suppl Table 2_Types of institutional settings.docx]

**Supplementary Table 2: Types of institutional settings in which participants practiced (n=1917)**

| Institutional Setting | n | % |
| --- | --- | --- |
| Private Practice or Self-Employed | 764 | 39.85% |
| Healthcare | 369 | 19.25% |
| Education | 239 | 12.47% |
| Community Mental Health Care | 147 | 7.67% |
| Nonprofit - Community Social Service Agency | 125 | 6.52% |
| Child Welfare | 49 | 2.56% |
| Government | 30 | 1.56% |
| Housing | 27 | 1.41% |
| Criminal Justice | 11 | 0.57% |
| Other | 156 | 8.14% |
| TOTAL | 1,917 | 100% |
