# Supplementary material for: The Impact of the COVID-19 Pandemic on Social Workers at the Frontline: A Survey of Canadian Social Workers
Source: Br J Soc Work. 2021 Jul 27:bcab158. doi: 10.1093/bjsw/bcab158 (PMC8406887; doi:10.1093/bjsw/bcab158)
Supplement: bcab158_Supplementary_Data [file bcab158_Supplementary_Data.zip › Suppl Table 1_Demographics of respondents.docx]

**Supplementary Table 1: Demographics of participants including age, gender, and ethnicity**

|  | % (n) |
| --- | --- |
| Gender* (n = 2,452) | |
|  | |
| Female | 82.95% (2,034) |
| Male | 14.36% (352) |
| Queer | 1.35% (33) |
| Non-Binary or Gender Fluid | 1.06% (26) |
| Age (n = 2,457) | |
| 18 to 24 | 1.79% (44) |
| 25 to 34 | 14.69% (361) |
| 35 to 44 | 21.57% (530) |
| 45 to 54 | 23.00% (565) |
| 55 to 64 | 23.85% (586) |
| 65 to 74 | 12.78% (314) |
| 75 and over | 2.32% (57) |
| Ethnicity (n = 2,357) | |
| White | 88.59% (2,088) |
| Black | 7.47% (176) |
| Indigenous | 4.16% (98) |
| Asian - South | 3.99% (94) |
| Asian - East | 2.12% (50) |
| Middle Eastern | 1.70% (40) |
| Latin American | 1.40% (33) |
| Asian – South East | 1.01% (24) |
|  |  |

*Participants also identified as transgender (n = 2), agender (n = 2), and two-spirit (n = 3).
